# Hsc70 Is a Novel Interactor of NF-kappaB p65 in Living Hippocampal Neurons

**DOI:** 10.1371/journal.pone.0065280

**Published:** 2013-06-07

**Authors:** Christin Klenke, Darius Widera, Thomas Engelen, Janine Müller, Thomas Noll, Karsten Niehaus, M. Lienhard Schmitz, Barbara Kaltschmidt, Christian Kaltschmidt

**Affiliations:** 1 Cell Biology, University of Bielefeld, Bielefeld, Germany; 2 Cell Culture Technology, University of Bielefeld, Bielefeld, Germany; 3 Proteome and Metabolome Research, University of Bielefeld, Bielefeld, Germany; 4 Institute of Biochemistry, Medical Faculty, Justus-Liebig-University, Giessen, Germany; 5 Molecular Neurobiology, University of Bielefeld, Bielefeld, Germany; Temple University, United States of America

## Abstract

Signaling via NF-κB in neurons depends on complex formation with interactors such as dynein/dynactin motor complex and can be triggered by synaptic activation. However, so far a detailed interaction map for the neuronal NF-κB is missing. In this study we used mass spectrometry to identify novel interactors of NF-κB p65 within the brain. Hsc70 was identified as a novel neuronal interactor of NF-κB p65. In HEK293 cells, a direct physical interaction was shown by co-immunoprecipitation and verified via *in situ* proximity ligation in healthy rat neurons. Pharmacological blockade of Hsc70 by deoxyspergualin (DSG) strongly decreased nuclear translocation of NF-κB p65 and transcriptional activity shown by reporter gene assays in neurons after stimulation with glutamate. In addition, knock down of Hsc70 via siRNA significantly reduced neuronal NF-κB activity. Taken together these data provide evidence for Hsc70 as a novel neuronal interactor of NF-κB p65.

## Introduction

The inducible transcription factor NF-κB is composed of dimeric DNA-binding subunits including p50, p52, c-Rel, RelB, and p65 (RelA). The most abundant NF-κB-heterodimer detected within the central nervous system (CNS) consists of p65 and p50 [1]–[3]. In its inactive form, NF-κB is kept in the cytoplasm by inhibitory IκB-proteins.

Within the CNS several crucial functions of NF-κB have been characterized in detail, such as the participation in neuroprotection, learning and memory formation [3]–[9]. NF-κB can be activated by a wide range of neuronal signals like neurotrophic factors, neurotransmitters or membrane depolarization [6].

We and others described that active NF-κB associates with microtubules via entry into the dynein/dynactin motor protein complex during its retrograde transport [10]–[12]. Moreover, in peripheral neurons NF-κB can translocate from activated synapse to the nucleus by associating with wild type Huntington protein, but not with mutant protein via association with importin α2 [13]. Importantly, the interaction of nuclear translocation signal with importin-α is essential for synapse-to-nucleus transport of NF-κB p65 [11] suggesting a huge regulatory impact of such direct interactions on the resulting signaling and transcriptional regulation.

Heat shock proteins (HSPs) were initially described as a large family of proteins mediating the cellular response to environmental stress such as elevated temperature, heavy metals or anoxia. However, they play also an important role in cell differentiation, proliferation and are implicated in tumor cell invasion. Within the CNS, HSPs have tremendous impact not only on protein folding but also on processes such as synaptic transmission, stress response, protein kinase-mediated signaling as well as cell death (reviewed in [14]). Interestingly, the Heat Shock Cognate 70 (Hsc70), the constitutively expressed form of HSP70, is localized specifically in synapses [15], [16] suggesting its involvement in synaptic signal transduction.

In this study we identified Hsc70 as a novel interaction partner of NF-κB using immunoprecipitation with subsequent mass spectrometry. In summary, we demonstrate that Hsc70 interacts directly with NF-κB-p65 in living hippocampal neurons and has a major impact on nuclear translocation and regulation of transcriptional activity via NF-κB in neuronal cells.

## Materials and Methods

### Ethics Statement

Prior to tissue isolation, mice were kept under specific pathogen free conditions as defined by the Federation European Laboratory Animal Science Association (FELASA) in the central animal facility of Bielefeld University. This study was carried out in strict accordance with the regulations of the governmental animal and care use committee, LANUV of the state North Rhine-Westphalia, (Düsseldorf, Germany). All animal tissue isolation procedures were approved by the Ethical Committee LANUV of the state North Rhine-Westphalia (Düsseldorf, Germany). All efforts were made to minimize suffering and animal number.

### Culture of Cell Lines

The adherent growing cell line HEK293FT was cultivated in DMEM (PAA, Colbe, Germany) supplemented with 200 µg/mL G418 (Sigma-Aldrich, Taufkirchen, Germany) in order to maintain the plasmid pCMVSPORT6TAg.neo. The medium was changed at least every three days. If confluent, the cells were split at a ratio of 1∶10 up to 1∶20.

### Astrocyte Cultures

Rat or mouse astrocytes were prepared from the cortex of postnatal day 1 (P1) Wistar rats or BL6 mice, after treatment with 1×Trypsin/EDTA (PAA). The astrocytes were washed with pre-warmed DMEM (37°C, PAA) and transferred to DMEM containing 2 mM L-glutamine, 100 U/ml penicillin and streptomycin and 10% fetal bovine serum (FCS, PAA). Cells were further cultured in a humidified incubator at 5% CO_2_. 1 day before hippocampi preparation the astrocytes were treated with 10 µg/ml mitomycin (Sigma-Aldrich) for 1.5 h. Directly before preparation of the hippocampi the astrocytes were transferred to pre-warmed Neurobasal medium (Invitrogen, Karlsruhe, Germany) supplemented with B27 supplement (Invitrogen), 2 mM L-glutamine (PAA), 100 U/ml penicillin (PAA) and 100 U/ml streptomycin (PAA).

### Hippocampal Neuron Cultures

Primary cultures of rat/mouse hippocampal neurons were prepared from the hippocampi of E18–E19 Wistar rat/BL6 mice embryos, after treatment with 1×Trypsin/ETDA (15 min, 37°C; (0.05%/0.002% in PBS) PAA). The hippocampi were then washed with pre-warmed DMEM (37°C) containing 10% FCS, to stop trypsin activity and transferred to pre-warmed DMEM supplemented with 2 mM L-glutamine, 100 U/ml penicillin, 100 U/ml streptomycin and 10% FCS. Cells were triturated with a fire-polished Pasteur pipette in this solution and afterwards plated on poly-D-Lysine (Sigma-Aldrich) coated coverslips at a density of 50000 cells/18 mm coverslip. The cultures were maintained in a humidified incubator at 5% CO_2_ for 60 min to allow the cells to adhere. After 1 h the neurons growing on coverslips were placed on top of mitomycine-treated astrocyte cultures and further cultivated at 37°C and 5% CO_2_.

### Anesthesia of Neuronal Activity for Baseline of Nuclear NF-κB

24 h prior to the experimental procedure, hippocampal neuron cultures were treated with 40 µM CNQX (Sigma-Aldrich), 100 µM APV (Sigma-Aldrich) and 10 µM nimodipine (Sigma-Aldrich) to establish a stable and low baseline of nuclear NF-κB.

### Pharmalogical Blockade of Hsc70

For pharmacological blockade of Hsc70 38 µg/ml deoxyspergualin ((DSG); Sigma-Aldrich)) was applied 60 min prior to the stimulation as previously described [17].

### Immunocytochemistry

Neurons were fixed using 4% paraformaldehyde (PFA) for 1 h at 4°C and permeabilized with 0.1% Triton X-100 in 1×PBS for 30 min at room temperature, followed by blocking using appropriate normal serum (5%, Jackson Immuno Research Laboratories). The primary antibodies [mouse monoclonal anti-α-tubulin (1∶100, Sigma-Aldrich), mouse anti- NF-κB p65 (1∶100, Santa Cruz Biotechnology Inc., Santa Cruz, California, USA)] were incubated for 1 h followed by labeling with secondary detection antibodies and counterstaining for nuclei using SYTOX green (1∶10000, Invitrogen, Karlsruhe, Germany). Images were collected using an Inverted Confocal Laser Scanning Microscope (LSM 510, Carl Zeiss) and analyzed with the ZEN2008 software (Carl Zeiss).

### *In situ* Proximity Ligation Assay

For *in situ* PLA, the Duolink II kit (Olink bioscience, Uppsala, Sweden) was used. Neurons were fixed using 4% PFA for 1 h at 4°C and treated with 1×PBS containing 0.1% Triton X-100. Blocking was performed using Duolink blocking solution in a pre-heated humidity chamber for 30 min at 37°C. Thereafter, the primary antibodies [mouse anti-HSC70 (1∶100, Biotrend, Cologne, Germany), rabbit anti-α-tubulin, mouse anti-β-III-tubulin (1∶100, Sigma-Aldrich), rabbit anti-NF-κB p65 (1∶100, Santa Cruz), mouse anti-NF-κB p50 (1∶50, Santa Cruz), rabbit anti-Nestin (1∶100, Sigma-Aldrich), mouse anti-Reelin (1∶100, Millipore, Schwalbach/Ts., Germany)] were immediately applied and incubated for 1 h at RT followed by incubation with the PLA-probes (1∶5, 2 h, RT)). Thereafter, cells were incubated with ligation solution (15 min at 37°C) followed by the amplification-polymerase solution (90 minutes at 37°C). Images were collected using Zeiss Axio Observer D1 (Carl Zeiss, Jena, Germany) and analyzed by using the AxioVision software (Zeiss). Quantification of PLA-signals (number of spots/cellular compartment) was performed using ImageJ-software (NIH).

### Immunoprecipitation for Mass Spectrometry

For immunoprecipitation 20 mg of brain extract in a volume of 1–2 ml were used. The protein complexes were cross-linked by addition of dithiobissuccinimidylpropionate (DSP) resulting in a final concentration of 0.5 mg/ml following by incubation on ice for 30 min. The cross-linking was stopped by addition of 25 mM Tris buffer (pH 8.0). The cross-linked protein was mixed with 50 µl protein G sepharose 4B fast flow (Sigma-Aldrich) and 30 µg of the anti p65/RelA antibody (sc-8008, Santa Cruz) or the isotype control (mouse monoclonal IgG_1_, MOPC 21, Sigma-Aldrich). The immunoprecipitates were slowly agitated for 2 h at 4°C. After immunoprecipitate-formation the samples were washed 3 times. During each washing step the samples were centrifuged for 1 min at 3000 g, the supernatants were discarded and 1 ml lysis buffer were added. After the last washing step the IPs were centrifuged and the pellet resolved in 30 µl 1×SDS sample buffer by heating at 60°C for 5 min. The supernatant was used for SDS gel electrophoresis and subsequent mass spectrometry analysis.

### Mass Spectrometry

Porcine brain extracts were immunoprecipitated with anti NF-κB p65 AB on protein G with isotype control. The IP were separated in a 1D SDS gel. Each lane (p65 precipitate and control) were cut into 36 slices and prepared for MS by tryptic digestion. All 36 slices were analyzed by MS (MALDI-TOF, Ultraflex extreme, Bruker, Bremen, Germany). Seven samples in the range of 95 to 60 and 27 to 24 kDa were additionally analyzed by LC-ESI-MS/MS (Thermo Scientific). The mass spectrometry peptide data were compared to the human protein data base from Uniprot using Mascot. Only proteins with a score >80 were regarded, if those proteins were absent in the corresponding isotype control.

### Immunoprecipitation (IP) for Western Blotting

HEK293FT cells were transfected with the expression constructs pcDNA3.1(+)c-myc-HSC70, pcDNA3.1(+)c-myc-HSC70mut, pEF-FLAGpGKpuro p65WT or pCMV c-myc-Iκε using Lipofectamine2000™ (Invitrogen) according to the manufactureŕs recommendation. For co-transfections equimolar ratios of the constructs were used. The cells were harvested 36 h after transfection and resuspended in 1 ml of lysis buffer (50 mM HEPES, 150 mM NaCl, 1% NP-40 (v/v), pH 7.5) supplemented with protease inhibitors (1 mM PMSF; 10 µg/ml leupetine, 10 µg/ml aprotinine, 1 µg/ml pepstatine and 10 mM NaF). Debris was separated by centrifugation for 10 min at 14000 g at 4°C. For IP 30 µl 50% protein A sepharose beads were washed once with 1 ml PBS. The beads, 900 µl supernatant and 1.0 µg/ml of rabbit anti-c-Myc antibody (Sigma-Aldrich) were incubated spinning head over tail for 2 h at 4°C or 37°C. If indicated 3.3 mg axon enriched porcine brain extract and 0.5 mg/ml DSP for crosslinking were added. The remaining cross-linker was quenched by addition of TrisHCl (pH8.0) to a final concentration of 25 mM and incubation for 15 minutes at 4°C. The beads were centrifuged at 12000 g for 1 min and washed with 1 ml lysis buffer containing 50 mM Tris instead of HEPES. After five washing steps with this lysis buffer and one with PBS the beads were eluted with 90 µl 1×SDS-sample buffer and subsequently loaded on SDS-Page for western blotting. The western blotting was performed using the following antibodies: rabbit polyclonal anti-Flag 1∶4000 (Sigma-Aldrich), rabbit polyclonal anti c-Myc 1∶2000 (Sigma-Aldrich) and goat anti-rabbit IgG H+L HRP 1∶4000 (Jackson Immuno Research Laboratories, Suffolk, UK).

### Tissue Extraction

Porcine brain tissue was sheared in an equivalent amount of lysis buffer in a blender on lowest level. One fraction of the hashed brain was used for extract directly; the other one was filtrated by a sieve, which enriches the more robust axon/myelin containing white tissue in the filter cake. The filter cake and the unseparated brain matter were separately mixed with the two fold amount of lysis buffer (50 mM HEPES, 15 mM NaCl, 1% NP-40 (v/v), pH 7.5, 1 mM PMSF). Afterwards both, whole brain and the filter cake were sheared by Ultra-Turrax T25 (Janke & Kunkel IKA Labortechnik, Staufen, Germany). After incubation (30 min on ice), the suspensions were cleared by centrifugation steps at 15000 g for 20 min and subsequently at 40000 g for 30 min. The protein solutions were directly used for IP.

### Determination of the Impact of Hsc70 on Nuclear Translocation of p65 and NF-κB Activity in HEK293 Cells

HEK293FT cells were co-transfected either with a combination of p65 FPred, mock vector and Hsc70-GFP expression constructs or with p65 FPred, mock and GFP using Lipofectamine. In additional approaches, cells were co-transfected with p65 FPred, IκB and Hsc70 GFP or p65 FP red, IκB and GFP. All approach were cultivated for 36 h, fixed and counter-stained with DRAQ5 (nuclei). The nucleus and the whole cell body were defined as regions of interest and their mean fluorescence was detected using confocal microscopy (Zeiss LSM) with subsequent image analysis in ImageJ software.

### Gene Silencing

The Ambion Silencer siRNA Construction Kit (Ambion, Austin, USA) was used to produce siRNAs against mouse Hsc70. The target sequences for Hsc70 knock down were identified, followed by Blast searches to ensure that the sequences did not contain significant homology to any other known genes. The following sequences were used for the knock down of Hsc70:

TCAGGTGTATGAAGGTGAA, GCAACCCTATCATTACCAA, ACAACCGAATGGTCAATCATT and GCACAGGAAAGGAGAACAA.

Cultured mouse astrocytes were transfected with 1 µg of the constructs cloned into pSilencer expression vector (Ambion) using Nucleofector II - device and Cell line-Nucleofection-Kit (Lonza, Verviers, Belgium) according to manufacturer’s protocol. After 48 h the cells were lysed and processed for western blot analysis using anti-Hsc70 antibody, whereas GAPDH served as loading control. Densitometric quantification (ratio Hsc70/GAPDH) was performed using ImageJ software. The construct inducing the strongest knock-down in astrocytes was used for the transfection of hippocampal neurons (GCAACCCTATCATTACCAA). Freshly isolated neurons were transfected with 1 µg of the construct using Nucleofector II. Anti-GFP siRNA served as control. After 48 h, transfected neurons were stimulated with glutamate as described above and processed for the experiments.

## Results

### Hsc70 is a Novel Interaction Partner of NF-κB

In this study, we analyzed potential novel interactors of NF-κB complexes acquired by co-precipitation with the NF-κB subunit p65 from neuronal extracts by a mass spectrometric analysis.

**Figure 1:**
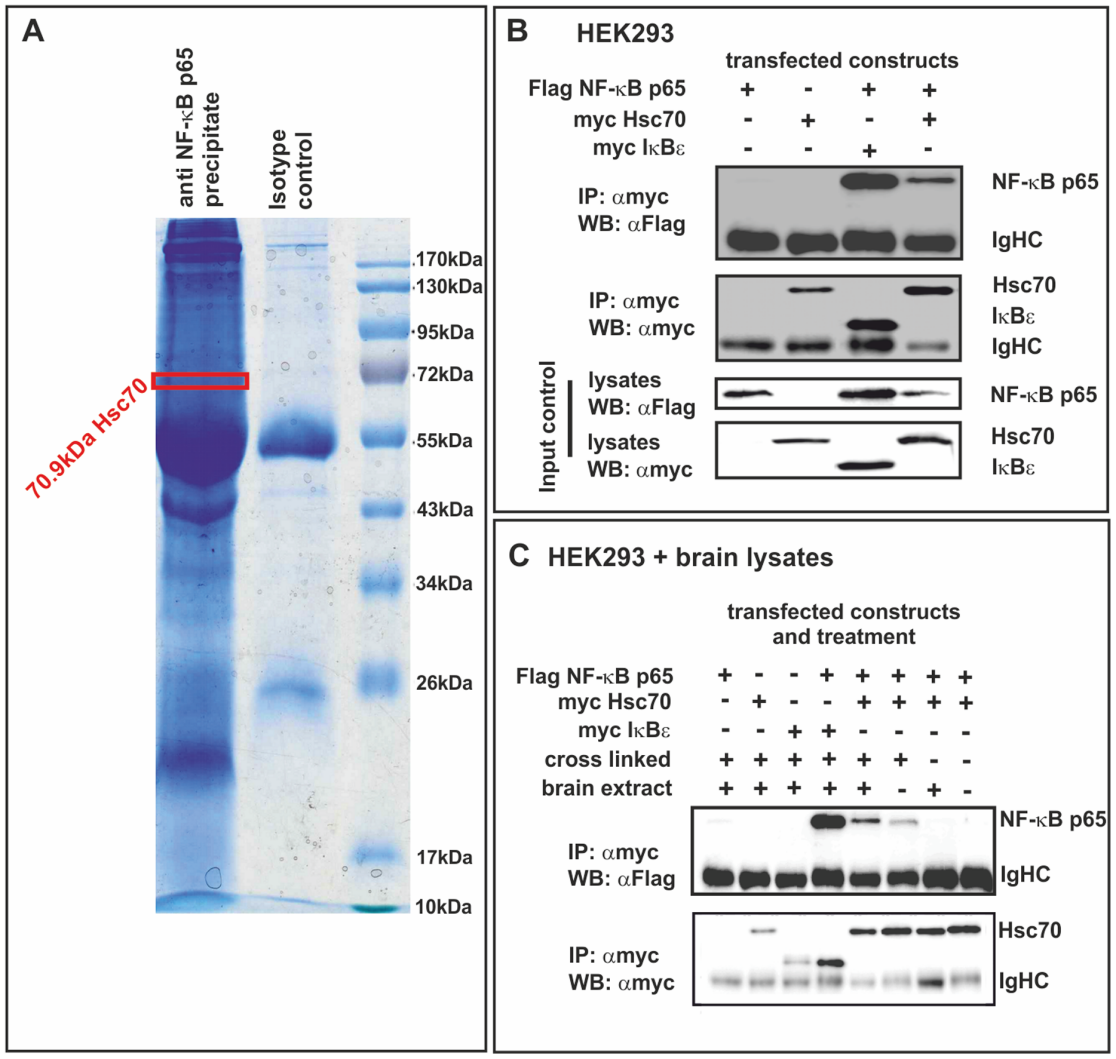
Hsc70 is a novel neuronal interaction partner of NF-κB. **A.** Porcine brain extracts were immunoprecipitated with anti NF-κB p65 antibody or isotype control on protein G sepharose in presence of cross-linker. The IP were separated in a 1D SDS gel. Each lane (p65 precipitate and control) were cut into 36 slices and prepared for MS by trypsin digestion. All 36 slices were analyzed by MS. Seven samples in range of 95 to 60 and 27 to 24 kDa were additionally analyzed by LC-ESI-MS/MS. The hits identified by MS included the heat shock cognate Hsc70 as a potential interaction partner of NF-κB p65. **B.** HEK293 co-transfected with p65-flag and Hsc70-myc or IκBε-myc were lysed followed by co-immunoprecipitation in presence of cross-linker using αmyc (IP) antibody with subsequent WB analysis. A clear interaction band (WB: αFlag) was detectable if myc-tagged IκBε and flag-tagged NF-κB p65 were co-transfected. Similarly, co-transfection of p65-flag and Hsc70-myc resulted in a clear interaction band (WB: αFlag), whereas no band was observed in negative controls (no p65-flag, or no IκBε-myc or Hsc70-myc). Lysates were used as input control. **C.** Neuronal proteins influence the interaction of NF-κB p65 with Hsc70. IP (αmyc) was performed in presence of cross-linker (DSP) and/or brain lysates with subsequent analysis by western blot. Interaction bands (WB: αFlag) were detectable in cross-linked samples for myc-tagged IκBε and flag tagged NF-κB p65 as well as for Hsc70-myc and NF-κB p65-flag. Combination of cross-linker and brain lysates resulted in stronger interaction band (WB: αFlag) for Hsc70-myc and NF-κB p65-flag. Without cross-linker no interaction bands was detectable.

As a source for neuronal NF-κB interactors we used porcine brains yielding high amounts of protein. MALDI-MS and LC-ESI-MS/MS revealed an interaction of p65 with compounds of the endocytosis network: clathrin and dynamin-1, microtubule subunits or associated proteins like beta 5-tubulin, tubulin alpha 6, beta actin, dihydropyrimidinase-related protein 2, neurofilament and light polypeptide (NEFL) and heat shock proteins HSP90 alpha class A and B (data not shown). Moreover, using MS we detected for the first time potential interaction of p65 with the protein chaperone heat shock cognate 70 kDa (Hsc70) alias HSPA8 (figure 1A). Since a high expression level of Hsc70 instead of HSP70 seems to be a hallmark of the nervous system [18], we hypothesized that Hsc70 may represent a novel neuronal interaction partner of NF-κB. The interaction of p65 and Hsc70 was further investigated by co-immunoprecipitation using anti-myc antibody and western blotting of flag-tagged proteins derived from lysates of HEK293 overexpressing p65-flag and Hsc70-myc or IκBε-myc as positive control (Figure 1B). A clear interaction band (WB: αFlag) was detected for Hsc70 and the positive control IκBε, whereas no band was observed in negative controls.

### Neuronal Proteins Influence the Interaction with NF-κB p65 and Hsc70

To address the question whether the presence of neuronal proteins influences the interaction discovered here, HEK293 cells were transiently co-transfected with flag-tagged p65 and myc-tagged Hsc70. After 36 h cells were harvested, lysed and processed for immunoprecipitation (IP). IP was performed using anti-myc-antibody in presence of cross-linker (DSP) and/or brain lysates. A clear interaction band for Hsc70-myc and p65-flag (WB: αFlag) was detected in presence of cross-linker (Fig. 1C). Moreover, brain lysates further increased the Hsc70-p65 interaction band (WB: αFlag) In contrast, without cross-linker or brain lysate no band was detectable (Fig. 1C).

### The Interaction of Hsc70 with NF-κB p65 is Lost after Mutation within the ATPase Domain of Hsc70 (F68 = >C)

**Figure 2:**
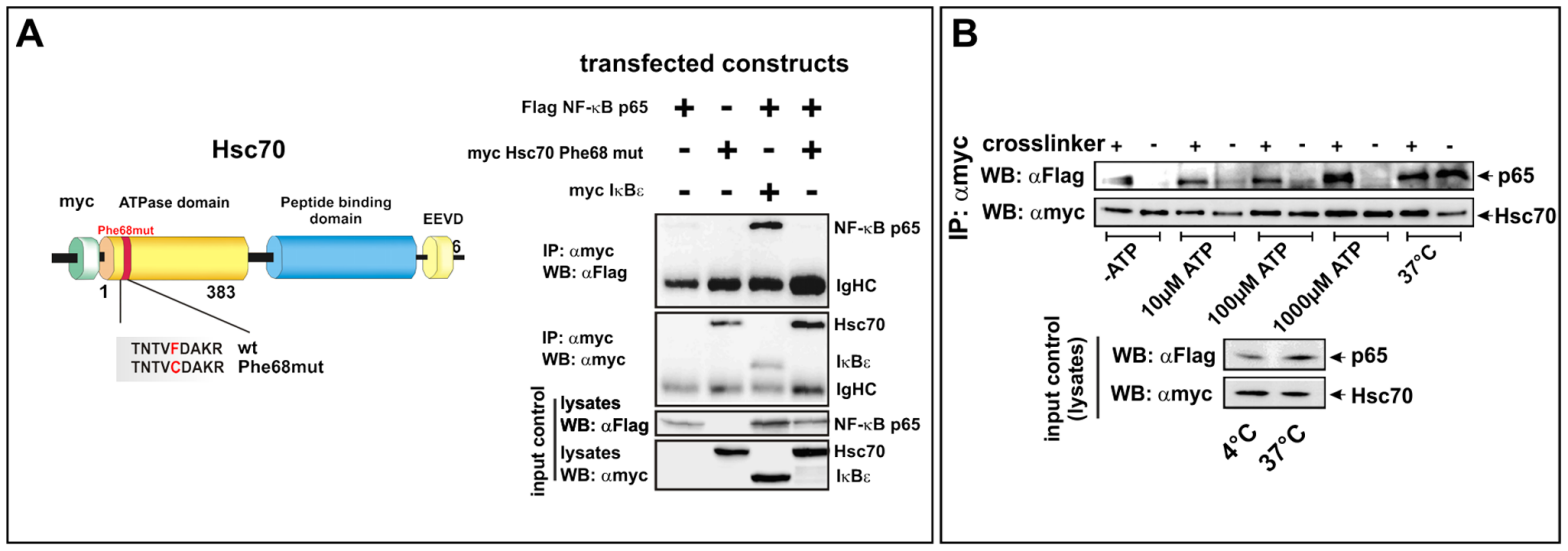
Interaction with NF-κB p65 is modulated by ATP and is lost after mutation of the ATPase domain of Hsc70. **A.** Hsc70 Phe68mut is not able to interact with NF-κB p65. Scheme: domain structure of the Hsc70-construct used for the experiments. The random mutant harbors a point mutation within the ATPase domain of Hsc70 at the position 68 (F = >C)**.** If myc-tagged Hsc70 Phe68mut was used in the IP (αmyc) no interaction band was detected for FLAG-tagged p65. Lysates were used as input control. **B.** In absence of cross-linker and ATP, Hsc70 shows no interaction with NF-κB p65 at 4°C. This interaction is moderately increased in presence of 10 µM and 100 µM ATP even without cross-linking. If the IP is performed at 37°C, the cross-link is dispensable even without ATP. Lysates were used as input control.

Due to the known function of Hsc70 in the nervous system acting as a clathrin uncoating ATPase during the anterograde axonal transport [19] we analyzed the potential impact of a mutation (F68 = >C) within the ATPase domain of Hsc70 on the interaction with p65. We created an Hsc70 mutant with a mutated ATPase domain (see scheme in Fig. 2A) to assess the ability of this mutant to interact with NF-κB p65. IP (αmyc-antibody) and a subsequent western blot developed with anti-Flag antibody using p65-flag and myc-tagged Hsc70 Phe68mut revealed no interaction band (Fig. 2A, WB: αFlag. Compare with the wild-type in Fig. 1B).

### The Interaction of Hsc70 and NF-κB p65 is Modulated by ATP and Depends on Temperature

Since Hsc70 dissociates clathrin coats from vesicles in an ATP-dependent manner [19], the potential influence of different ATP concentrations on the interaction was investigated. In parallel, the influence of temperature on the interaction was determined (see Fig. 2B). Here, an interaction between p65-flag and Hsc70-myc was detected in all cross-linked samples. Moreover, samples without cross-linker showed weak interaction bands in presence of 10 µM as well as 100 µM ATP. If the IP was performed at 37°C, an interaction band was detected in absence of cross-linker.

### p65 Interacts with Hsc70 in Cultured Hippocampal Neurons

**Figure 3:**
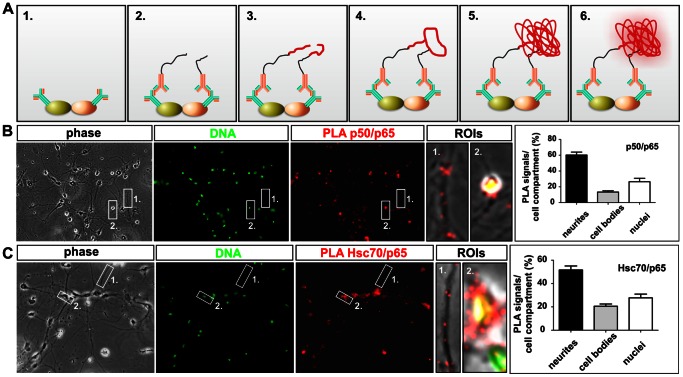
*in situ* Proximity Ligation Assay (PLA) reveals direct interaction of Hsc70 with NF-κB p65. **A.** Principle of PLA. **(**1) Incubation with primary antibodies against the two epitopes of interest. (2**)** Incubation with PLA probe MINUS and PLA probe PLUS (secondary antibodies conjugated with oligonucleotides). (3) Hybridization of the oligonucleotides with the PLA probes. (4) Ligation. (5) Rolling-circle amplification. (6) Detection. **B.** *in situ* PLA performed using p65 and p50 probes revealed the majority of interaction signals within the neurites (ROI 1) and nuclei (ROI 2) instead of cell bodies in hippocampal neurons (see also quantification on the right-hand side). **C.** For p65 and Hsc70, interaction signals were detected in neurites (ROI 1) and nuclei (ROI 2) with subcellular distribution similar to p65/p50 PLA-probes.

To find out if there is a direct interaction between the Hsc70 and p65 in hippocampal neurons we used the *in situ* Proximity Ligation Assay (PLA) (for principle see Figure 3A). *In situ* PLA is a simple technique for detection of direct protein-protein interactions using two primary antibodies, each directed against one of the targets of interest [20].

As a positive control we applied *in situ* PLA for visualization of the interaction between the NF-κB subunits p50 and p65 within the nuclei (Fig. 3B, ROI 2), cell bodies and neurites (Fig 3B, ROI 1) of hippocampal neurons. Similarly, the use of p65 and the Hsc70 as PLA-probes resulted in clear interaction signals with similar subcellular distribution (see Fig 3 ROIs and quantification). The largest fractions of interaction signals for p50/p65 as well as for Hsc70/p65 were observed within neurites and nuclei of hippocampal neurons. In negative controls without primary antibodies as well as in combinations of p65 antibody and unrelated control antibodies no signal was detected (data not shown).

### Hsc70 Inhibition Reduces the Nuclear Translocation of NF-κB in Hippocampal Neurons

**Figure 4:**
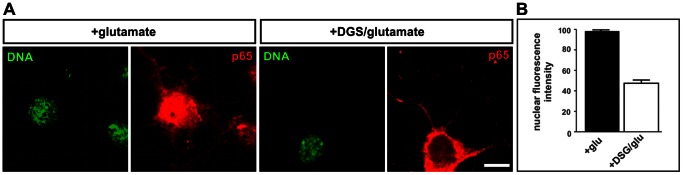
Hsc70-inhibition decreases the nuclear translocation of NF-κB in neurons. **A.** Hippocampal neurons were treated with deoxyspergualin (DSG) followed by immunocytochemical staining for NF-κB p65. The treatment with DSG resulted in reduced nuclear p65. Scale bar represents 10 µm. **B.** Quantification of nuclear p65 after treatment with DSG. In neurons treated with DSG and glutamate, the nuclear NF-κB p65 significantly decreased to 53% compared to glutamate-stimulated controls. p value <0.0001.

To assess whether the interaction of p65 and Hsc70 is required for the nuclear translocation of NF-κB in neuronal cells, hippocampal neurons were treated with the Hsc70 inhibitor DSG followed by stimulation with glutamate. Immunohistochemical analysis after treatment with DSG resulted in significantly reduced nuclear p65 after glutamate stimulation (∼53%) compared to DSG-untreated, glutamate-stimulated controls (Fig. 4a).

### Overexpression of Hsc70 Results in Increased Nuclear p65 and NF-κB Activity

**Figure 5:**
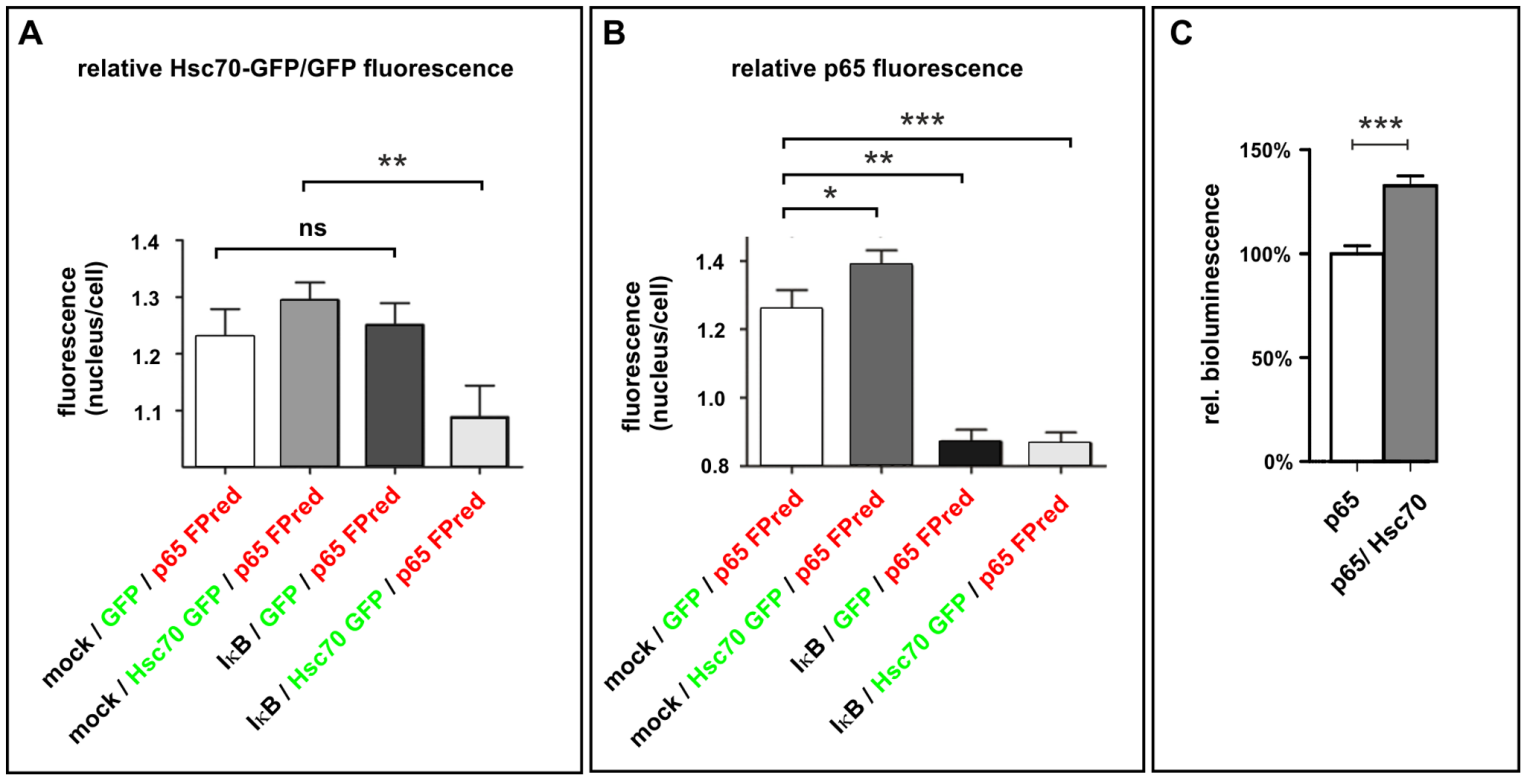
Overexpression of Hsc70 increases nuclear p65 and elevates the activity of NF-κB. **A.** HEK293FT cells were transfected with expression vectors as indicated. An inactive state of NF-κB (baseline) was achieved by co-transfection of IκB expression vector. The nuclei and the whole cell body were defined as regions and their mean fluorescence was measured using ImageJ-software. Co-expression of IκB significantly reduces nuclear Hsc70 GFP, whereas no effect on the nuclear localization of GFP was observed. **B.** HEK293FT cells were transfected as indicated in A. Co-expression with Hsc70 GFP significantly increased nuclear p65-FPred compared to co-expression with GFP. No increase of nuclear FPred p65 was observed if additional IκB was expressed (data not shown). **C.** Luciferase reporter assay revealed a significant increase of NF-κB activity in HEK293 cells co-transfected with Hsc70 and p65 (+32%), compared to cells over-expressing p65 alone.

Recently, it has been demonstrated, that up-regulation of the non-constitutive HSP70 results in increased nuclear translocation of p65 [21]. Therefore, we hypothesized that Hsc70 may affect the nuclear import and the subsequent transcriptional activity of NF-κB in a similar manner. Here, HEK293FT cells were transfected with an FPred-p65 expression construct and in part with an Hsc70-GFP or a GFP expression vector, fixed and counter-stained with DRAQ5 to visualize the nuclei. An inactive state of NF-κB (baseline) was achieved by co-transfection of the IκB. The nuclei and the whole cell body were defined as regions and their mean fluorescence was measured using ImageJ-software. We were able to show that co-expression of IκB significantly reduces nuclear Hsc70-GFP, whereas no effect on the nuclear localization of GFP was observed (figure 5). Furthermore, co-expression with Hsc70-GFP significantly increased nuclear FPred-p65 compared to co-expression with GFP. In addition, luciferase reporter assay revealed a significant increase of NF-κB activity in cells co-transfected with Hsc70 and p65 (+32%), compared to cells over-expressing p65 alone (figure 5C).

#### Pharmacological blockade of Hsc70 leads to decreased nuclear interaction with p65

**Figure 6:**
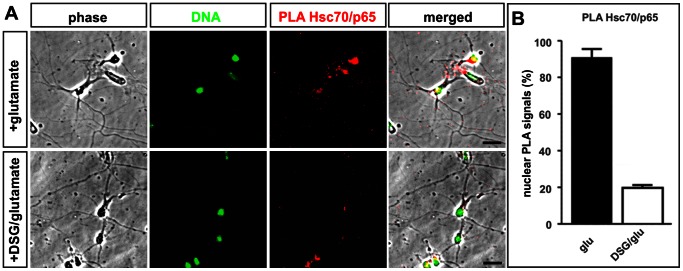
The blockade of Hsc70 reduces the level of interaction with p65. **A.** Using *in situ* PLA, we investigated the influence of the Hsc70 inhibiting drug DSG on the interaction with p65. Treatment of hippocampal neurons with DSG resulted in reduced nuclear interaction signals compared to controls. Scale bar represents 20 µm. **B.** Quantification of interaction between Hsc70/p65 after pharmacological treatment. The treatment of hippocampal neurons with DSG resulted in markedly reduced amount of nuclear interaction signals compared to controls stimulated with glutamate only.

Using *in situ* PLA, we investigated the influence of Hsc70 enzymatic activity on its ability to interact with p65 in hippocampal neurons. Treatment with DSG reduced the degree of interaction as demonstrated by PLA (Figure 6A). Quantification of nuclear interaction signals visualized using *in situ* PLA revealed significantly decreased nuclear interaction in DSG-treated hippocampal neurons compared to controls (Figure 6B).

### siRNA Mediated Knock-down of Hsc70 Results in Significantly Reduced NF-κB Activity

**Figure 7:**
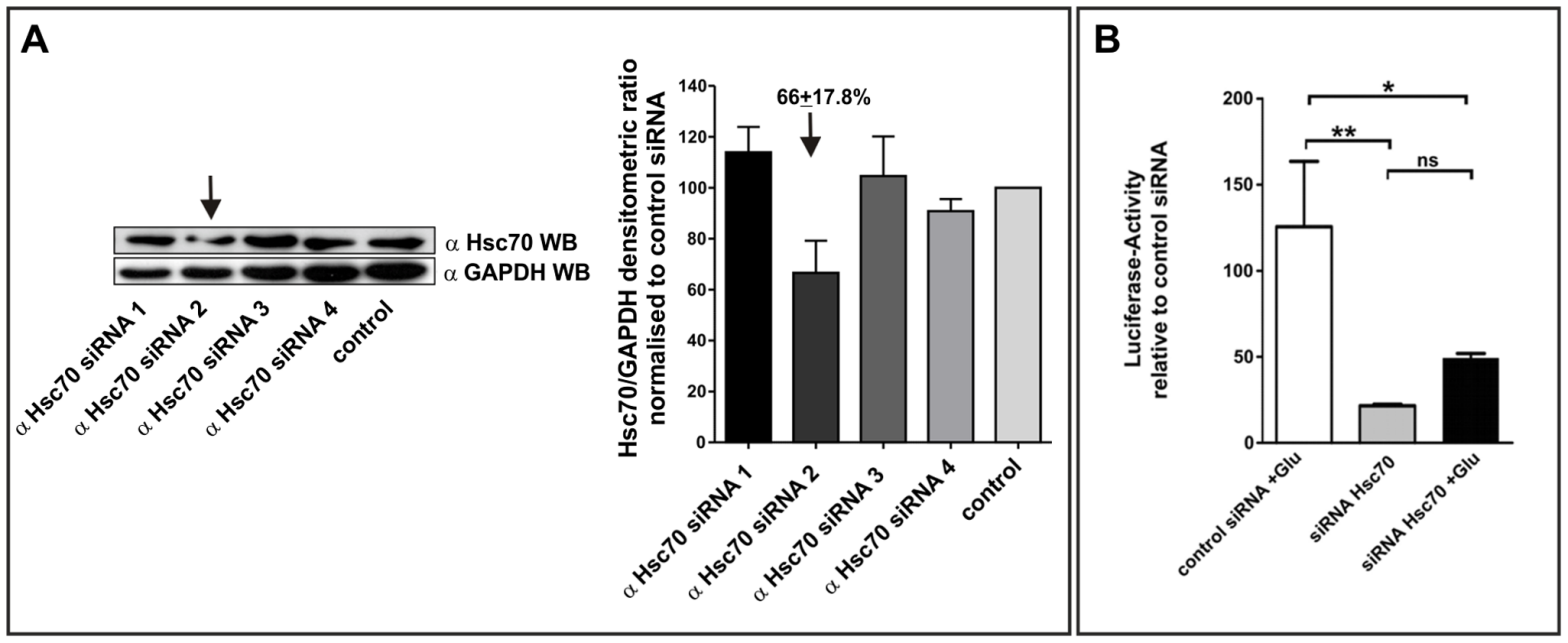
siRNA mediated knock-down of Hsc70 results in significantly reduced NF-κB activity. **A.** Screening for functional- and non-functional constructs in mouse astrocytes. Cells were transfected with siRNA expression constructs, cultivated for 48h and processed for western blot analysis using anti-Hsc70 antibody. GAPDH was used as control. The construct inducing the strongest knock-down in astrocytes (arrow, 66% of the control) was used for the transfection of hippocampal neurons. **B.** Hippocampal neurons were transfected with luciferase reporter plasmids and αHsc70 siRNA or control siRNA processed for NF-κB dependent luciferase activity assay. A knock-down of Hsc70 resulted in significantly reduced NF-κB activity compared to control. Note that neurons transfected with αHsc70 siRNA showed low NF-κB activity even after glutamate stimulus. p value <0.0001.

To further assess the influence of the interaction on NF-κB-activity, we designed siRNA-constructs to induce a knock-down of Hsc70. After screening for functional- and non-functional constructs in glial cells (see Figure 7A), hippocampal neurons were co-transfected with NF-κB luciferase reporter plasmid and the most effective anti Hsc70 siRNA (siRNA construct 2, 66% of the control siRNA level) or control siRNA followed by detection of NF-κB-dependent luciferase activity. Here, we demonstrated that knock-down of Hsc70 resulted in significantly reduced NF-κB activity compared to neurons transfected with control siRNA (Figure 7B). Remarkably, knock-down of Hsc70 resulted in reduced NF-κB activity even after glutamate stimulation.

## Discussion

In this study, we demonstrate for the first time that the protein chaperone Hsc70 directly interacts with NF-κB in living hippocampal neurons and has a major impact on the transcriptional activity of NF-κB.

We and others showed that NF-κB is transported from the synapse back to the nucleus via the minus-end motor protein dynein along the microtubuli [3], [10] [11], [12]. Mikenberg et al. suggested that the nuclear translocation signal interacting with importin-α is essential for synapse-to-nucleus transport of NF-κB p65 [11].

Using immunoprecipitation with subsequent analysis via mass spectrometry we identified Hsc70 as a neuronal interaction partner of NF-κB p65. Remarkably, the inducible Hsc70-homologue HSP70 is expressed only at low levels in the CNS, whereas Hsc70 seems to be expressed at high levels in mature neurons and the developing brain [18]. In neuronal cells, Hsc70 is localized predominantly within synapses suggesting that Hsc70 might participate in synaptic transmission [15], [16].

In this study, the over-expression of Hsc70 led to increased nuclear p65, whereas the inhibition of p65 by over-expression of IκB resulted in decreased nuclear Hsc70 (figure 5). Consequently, the nuclear translocation Hsc70 and p65 might occur as a protein/protein complex. This is in general accordance with findings described for the inducible form HSP70, whose up-regulation was reported to induce nuclear translocation of p65 in rat liver cells [21].

It has been suggested that during the anterograde axonal transport Hsc70 may act as a clathrin uncoating ATPase participating in the kinesin mediated plus-end transport [19]. In our study, we created an Hsc70 mutant carrying a point mutation in the ATPase domain (position 68 (F = >C)) resulting in a loss of interaction with NF-κB p65. Moreover, we show that this interaction is moderately increased in presence of 10 µM and 100 µM ATP. This finding correlates with findings on the GR transport complex, in which the inducible Hsc70-homologue HSP70 binds the cargo independent from the type of nucleotide bound (ADP or ATP). The further assembly of the complex, which grants stable interaction, needs HSP70s ATPase activity [22]. Demonstrably, the inhibitor of Hsc70 DSG does not compete for peptide binding, but blocks a c-terminal regulatory motif which appears to regulate the ATPase activity and thus the ability to interact with protein substrates [23]. However, since we tested the impact of the mutation within the ATPase domain of Hsc70 in the context of interaction with NF-κB p65, the exact role of Hsc70 ATPase activity in translocation and transcriptional activity of NF-κB is clear and should be investigated in future studies.

Here, the pharmacological blockade of Hsp70 via DSG resulted in diminished nuclear translocation (Figure 4) and reduced interaction of Hsc70 with p65 in the nuclei of hippocampal neurons (Figure 6). In addition to the pharmacological approach, siRNA-mediated knock-down of Hsc70 is sufficient for strongly reduced NF-κB activity, as demonstrated by a reporter gene assay (Figure 7).

This is in general accordance with the study by Tepper et al., which showed that DSG, a clinically used potent immunosuppressive agent (Gusperismus, Bristol-Myers Squibb or Spanidin, Nordic Pharma Group), can suppress NF-κB activity in pre-B-cells [24].

Although one of the best characterized functions of heat shock proteins is the protein folding and mediation of degradation of misfolded proteins, the predominant localization of Hsc70-p65-complexes in neurites and nuclei suggests further regulatory role of Hsc70 in neurons. This and the synaptic localization of both - p65 and Hsc70, suggest an additional regulatory function which may be achieved via participation in synapse-to-nucleus transport similar to mechanism described for the retrograde transport of GR in neurons [25].

This finding might hint to potential neurological side-effects of Gusperimus (Spanidin) in patients, which might be explained by inhibiting the neuroprotective NF-κB signaling.

## References

[1] Kaltschmidt C, Kaltschmidt B, Baeuerle PA (1993) Brain synapses contain inducible forms of the transcription factor NF-kappa B. Mech Dev. 43: 135–147. 10.1016/0925-4773(93)90031-r 8297787

[2] Kaltschmidt C, Kaltschmidt B, Neumann H, Wekerle H, Baeuerle PA (1994) Constitutive NF-kappa B activity in neurons. Mol Cell Biol 14: 3981–3992. 8196637 10.1128/mcb.14.6.3981-3992.1994 PMC358764

[3] Meffert MK, Chang JM, Wiltgen BJ, Fanselow MS, Baltimore D (2003) NF-kappa B functions in synaptic signaling and behavior. Nat Neurosci 6: 1072–1078. 12947408 10.1038/nn1110

[4] Mattson MP, Culmsee C, Yu Z, Camandola S (2000) Roles of nuclear factor kappaB in neuronal survival and plasticity. J Neurochem 74: 443–456. 10646495 10.1046/j.1471-4159.2000.740443.x

[5] Fridmacher V, Kaltschmidt B, Goudeau B, Ndiaye D, Rossi FM, et al (2003) Forebrain-specific neuronal inhibition of nuclear factor-kappaB activity leads to loss of neuroprotection. J Neurosci 23: 9403–9408. 14561868 10.1523/JNEUROSCI.23-28-09403.2003 PMC6740573

[6] Kaltschmidt B, Widera D, Kaltschmidt C (2005) Signaling via NF-kappaB in the nervous system. Biochim Biophys Acta 1745: 287–299. 15993497 10.1016/j.bbamcr.2005.05.009

[7] Meffert MK, Baltimore D (2005) Physiological functions for brain NF-kappaB. Trends Neurosci 28: 37–43. 15626495 10.1016/j.tins.2004.11.002

[8] Mattson MP, Meffert MK (2006) Roles for NF-kappaB in nerve cell survival, plasticity, and disease. Cell Death Differ 13: 852–860. 16397579 10.1038/sj.cdd.4401837

[9] Kaltschmidt B, Kaltschmidt C (2009) NF-kappaB in the nervous system. Cold Spring Harb Perspect Biol 1: a001271. 20066105 10.1101/cshperspect.a001271 PMC2773634

[10] Wellmann H, Kaltschmidt B, Kaltschmidt C (2001) Retrograde transport of transcription factor NF-κB in living neurons. J Biol Chem 276: 11821–11829. 11096106 10.1074/jbc.M009253200

[11] Mikenberg I, Widera D, Kaus A, Kaltschmidt B, Kaltschmidt C (2007) Transcription factor NF-kappaB is transported to the nucleus via cytoplasmic dynein/dynactin motor complex in hippocampal neurons. PLoS ONE 2: e589. 17622342 10.1371/journal.pone.0000589 PMC1899224

[12] Shrum CK, Defrancisco D, Meffert MK (2009) Stimulated nuclear translocation of NF-kappaB and shuttling differentially depend on dynein and the dynactin complex. Proc Natl Acad Sci U S A 106: 2647–2652. 19196984 10.1073/pnas.0806677106 PMC2650318

[13] Marcora E, Kennedy MB (2010) The Huntington’s disease mutation impairs Huntingtin’s role in the transport of NF-kappaB from the synapse to the nucleus. Hum Mol Genet 19: 4373–4384. 20739295 10.1093/hmg/ddq358 PMC2957321

[14] Stetler RA, Gan Y, Zhang W, Liou AK, Gao Y, et al (2010) Heat shock proteins: cellular and molecular mechanisms in the central nervous system. Prog Neurobiol 92: 184–211. 20685377 10.1016/j.pneurobio.2010.05.002 PMC2939168

[15] Suzuki T, Usuda N, Murata S, Nakazawa A, Ohtsuka K, et al (1999) Presence of molecular chaperones, heat shock cognate (Hsc) 70 and heat shock proteins (Hsp) 40, in the postsynaptic structures of rat brain. Brain Res 816: 99–110. 9878698 10.1016/s0006-8993(98)01083-x

[16] Bharadwaj S, Ali A, Ovsenek N (1999) Multiple components of the HSP90 chaperone complex function in regulation of heat shock factor 1 In vivo. Mol Cell Biol 19: 8033–8041. 10567529 10.1128/mcb.19.12.8033 PMC84888

[17] Sugawara A, Torigoe T, Tamura Y, Kamiguchi K, Nemoto K, et al (2009) Polyamine compound deoxyspergualin inhibits heat shock protein-induced activation of immature dendritic cells. Cell Stress Chaperones 14: 133–139. 18686015 10.1007/s12192-008-0064-y PMC2727995

[18] D’Souza SM, Brown IR (1998) Constitutive expression of heat shock proteins Hsp90, Hsc70, Hsp70 and Hsp60 in neural and non-neural tissues of the rat during postnatal development. Cell Stress Chaperones 3: 188–199. 9764759 10.1379/1466-1268(1998)003<0188:ceohsp>2.3.co;2 PMC312963

[19] de Waegh S, Brady ST (1989) Axonal transport of a clathrin uncoating ATPase (HSC70): a role for HSC70 in the modulation of coated vesicle assembly in vivo. J Neurosci Res 23: 433–440. 2475643 10.1002/jnr.490230409

[20] Soderberg O, Gullberg M, Jarvius M, Ridderstrale K, Leuchowius KJ, et al (2006) Direct observation of individual endogenous protein complexes in situ by proximity ligation. Nat Methods 3: 995–1000. 17072308 10.1038/nmeth947

[21] Dokladny K, Lobb R, Wharton W, Ma TY, Moseley PL (2010) LPS-induced cytokine levels are repressed by elevated expression of HSP70 in rats: possible role of NF-kappaB. Cell Stress Chaperones 15: 153–163. 19551494 10.1007/s12192-009-0129-6 PMC2866987

[22] Morishima Y, Kanelakis KC, Murphy PJ, Shewach DS, Pratt WB (2001) Evidence for iterative ratcheting of receptor-bound hsp70 between its ATP and ADP conformations during assembly of glucocorticoid receptor.hsp90 heterocomplexes. Biochemistry 40: 1109–1116. 11170435 10.1021/bi002399+

[23] Nadler SG, Dischino DD, Malacko AR, Cleaveland JS, Fujihara SM, et al (1998) Identification of a binding site on Hsc70 for the immunosuppressant 15-deoxyspergualin. Biochem Biophys Res Commun 253: 176–180. 9875240 10.1006/bbrc.1998.9775

[24] Tepper MA, Nadler SG, Esselstyn JM, Sterbenz KG (1995) Deoxyspergualin inhibits kappa light chain expression in 70Z/3 pre-B cells by blocking lipopolysaccharide-induced NF-kappa B activation. J Immunol 155: 2427–2436. 7650374

[25] Galigniana MD, Harrell JM, Housley PR, Patterson C, Fisher SK, et al (2004) Retrograde transport of the glucocorticoid receptor in neurites requires dynamic assembly of complexes with the protein chaperone hsp90 and is linked to the CHIP component of the machinery for proteasomal degradation. Brain Res Mol Brain Res 123: 27–36. 15046863 10.1016/j.molbrainres.2003.12.015

